# Albuminuria

**Published:** 1908-02

**Authors:** Edgar Ballenger

**Affiliations:** Atlanta, Ga., Lecturer on Genito-urinary Diseases, Atlanta School of Medicine


### THE DIAGNOSIS OF KIDNEY DISEASES WITH SPECIAL REFERENCE TO THE SIGNIFICANCE OF SLIGHT ALBUMINURIA.*

BY EDGAR BALLENGER, M. D., ATLANTA, GA.

LECTURER ON GENITO-URINARY DISEASES, ATLANTA SCHOOL OF MEDICINE.

The scope of this paper is limited to a consideration of certain phases of albuminuria and its bearing upon the diagnosis in the obscure, rather than the clearly defined cases. There is little doubt in advanced conditions where there are casts, albumin, edema, high arterial tension, etc., but we often feel uncertain as to the diagnosis and prognosis if there are only slight evidences of kidney disease, such as a trace of albumin or orthostatic albuminuria and a few casts.

When Richard Bright first called attention to the significance of albumin in the urine, the crude methods then used in testing it prevented the detection of small amounts of proteid that we now find so frequently and have so much difficulty in correctly interpreting. Hasting and Hoobler have made a large number of careful examinations, and have found that the cases of well-defined Bright's disease, in which the symptoms and physical signs pointed to a renal lesion, include only 12.59 per cent. of the patients with albuminuria. Nucleoalbumin was frequently found, and should always be differentiated from serum albumin.

Albumin in urine may be renal or extrarenal in origin. By renal, we mean that it passes through the kidneys with the urine, and may be due either to a local lesion in the kidney, or to a systemic condition, which, by general changes throughout the body, or by toxins circulating in the blood, injure to some extent the renal structure, and allow the escape of albumin. Extrarenal albumin may come from any part of the genito-urinary tract below the kidneys, and is a contamination of the urine with pro-

*Read before the Fulton County Medical Society, October 3, 1907, and reprinted from the Medical Record Nov. 30, 1907.

teid substances, for which we should be alert constantly, so as to avoid the error of mistaking it for renal albumin.

The commoner proteid substances that are found in the urine are serum albumin, serum globulin, nucleoalbumin and albumose. Serum albumin is the most important of these as a positive finding, and suitable tests should be made to eliminate the other proteids. Cohnheim's method of "salting" the urine with a saturated solution of sodium chloride, over acidulation with 3 to 5 drops of 50 per cent. acetic acid and boiling, will throw down the proteids commonly found in the urine. Nucleoalbumin is not precipitated by heat and acetic acid, though in highly salted urine; consequently a cloudiness with this test usually means serum albumin (Hasting).

Albumose frequently has its origin in an inflamed prostate gland or seminal vesicle. The proteid substances that are secreted in the prostatic discharge are digested by a ferment called leucoprotease, which is liberated from the disintegrating pus cells, and only acts in a faintly alkaline medium (Opie & Barker). This albumose is not precipitated by boiling, nor does it give the cloudiness at about 60 degrees C., as does Bence-Jones albumose. When precipitated with cold citric and picric acid, a sediment is formed, but will disappear when heated, and again return when allowed to cool. If serum albumin or nucleoalbumen be present, it only partially clears when heated, and, if the test tube is placed in a boiling water bath, these albumins will settle to the bottom, and when it is allowed to cool, the albumose is deposited in another layer upon it, a distinct line of demarcation between them being perceptible.

Bence-Jones albumose gives a cloudiness below the boiling point, but when further heated, clears. If stratified upon hydrochloric acid, it will also cause the appearance of a white ring at the zone of contact. The other proteids do not re-act to this test. (Bradshaw).

The acid secretion in the vagina prevents the digestive action of the leucoprotease which is liberated from the pus cells, and the proteid discharge is not changed into albumose, but is converted into an acid albuminate which cannot be detected by the ordinary heat test, and when precipitated will not clear up again as albumose does when reheated.

A prostatic abscess draining into the urethra will give large quantities of serum albumin and globulin at first, and later albumose. The presence of pus and albumin in such amounts at once suggest an abscess of the kidney. In a previous paper, I reported the history of a patient with an insidious prostatic abscess with urine exactly simulating a renal abscess.

Having found that we are dealing with albumin from the kidney, an even more difficult task is to determine its significance. It may be orthostatic in variety, as described years ago by our ex-president, Dr. A. W. Stirling, under the name postural albuminuria, as it only appears when the patient assumes an upright position. This form of albuminuria may, or may not be attended with tube casts, and may or may not be followed by serious kidney disease. As a rule, however, it is the least harmful, and a rather common form of albuminuria. It is known that more urea is eliminated when a patient is in the upright position, and that the urine has a higher specific gravity and is more highly colored, than when reclining. Why this should be the case we are unable to say. It has been assumed that the escape of albumin from the kidneys, when the patient is standing, is due to certain changes in the blood pressure, or to a paresis of the vasomotor system of the kidney.

The experiments of Lemoissier have shown that standing lowers the secretion of water by the kidneys in all individuals, but this diminution of water was found to be greater in those in whom the kidneys were diseased, and the difference was sufficiently marked and constant to indicate that excessive orthostatic oliguria is a very delicate sign of defective renal secretion. Concerning an increase in the albumin in the urine when patients with an undoubted nephritis assume an upright position, very little has been written, but from a consideration of the facts pertaining to orthostatic albuminuria, I feel confident that such a condition exists.

Fox, following the work of Wright, thought the trouble was due to a lessened coagulability of the blood, and gave patients calcium lactate, and again made tests when the coagulability had been increased, and claimed that when the albumin was purely functional it would disappear after the calcium lactate had been given, while if there be an organic renal lesion, the albumin will not be affected. I have tried this test on a number of patients, but have had no very conclusive results. One patient, with only a few hyaline casts and an orthostatic albuminuria, before taking the calcium lactate, had an enormous increase in the casts on the day following, and as many as forty were counted in one field with the low-power lens in a specimen from urine which had not been centrifugated, but had only been standing in a glass for about thirty minutes. With this increase in casts there was a diminution in the quantity of the albumin.

Simidar has made a number of experiments on animals and found that physiological (?) albuminuria occurs in them, as well as in man.

Collier, among whose duties is the examination of students at Oxford University, has been in the habit of advising students who have albuminuria after exercise to give up competitions involving great muscular strain. In 1906, however, he made systematic examinations of all the men training for boat racing and found that, without exception, every man who rowed the full course, passed albumin in his urine, and at least half passed it in considerable quantity. The same was true of running men, and has been observed by a number of previous investigators. Collier no longer holds that such men should give up hard athletic competitions.

Heubner has recently reported the post-mortem findings of a child in whom orthostatic albuminuria had been observed for a year and a half previous to death, which was caused by a brain tumor. The kidneys were found practically normal, the very slight changes being too trivial to be considered, and were thought to be due to the long death agony. A small tuberculous area was found at the apex of one lung, which tends to confirm the view held by some observers, that postural albuminuria is a "pre-tuberculous" phenomenon.

Examples of orthostatic albuminuria have been found to precede certain well-recognized forms of nephritis, and albuminuria in convalescents from scarlet fever, has finally changed to the orthostatic form.

Bruas has reported the history of a patient with intermittent orthostatic albuminuria during attacks of malaria. The observer was unable to say that malaria was the cause of the albuminuria, but asserts that it was undoubtedly a factor in producing it. In this connection, I desire to report the history of a patient of my own with syphilis, in whom orthostatic albuminuria was strikingly demonstrated. There were a few mucous patches in his mouth and he suffered with severe nocturnal headache. Syphilis had been contracted ten months previously and had received very little treatment. How long the albuminuria had existed, I am unable to say. There were a few hyaline and granular casts. Under anti-syphilitic treatment with iodide of potash and intramuscular injections of salicylate of mercury, the headache ceased immediately, the mucous patches were well in a week and the urine became practically normal within a month, but the albumin showed a tendency to recur at intervals during the next two months. The casts disappeared entirely. No etiological factor other than syphilis could be assigned as the cause of this patient's orthostatic albuminuria, and this view seems confirmed by the fact that under treatment for this disease it cleared up along with the obvious syphilitic symptoms, and again returned after the patient had discontinued treatment for four months while out of the city. It is my opinion that if left untreated, this condition will develop into a true syphilitic nephritis.

Tunis wrote letters to a number of the leading men of this country for their opinion regarding the significance of small quantities of albumin in the urine, and, from their replies and from his own experience, he has reached the conclusion that the mortality must necessarily be higher in these individuals than in those who do not show this abnormality.

Casts have about the same significance as the renal albumin. There are many drugs that cause their appearance, especially the hyaline variety. Severe muscular exertions and over indulgence in certain forms of food and alcoholics also produce them where there is no nephritis. Merely their presence in a single specimen of urine, which has been allowed to stand and the upper portion decanted off and the lower part carefully centrifugated, can not be taken in itself as evidence of Bright's disease—even if a few granular casts are found. When irritated, the nonnephritic kidney seems capable of producing them in larger numbers than can the diseased kidney—especially the interstitial nephritis in patients of advanced age (Emerson).

*Conclusions.*—In doubtful cases, no positive opinion should be expressed until many specimens of urine have been examined, for it is the persistence of renal albumin and casts that is of more serious import than is their occasional presence in considerably larger quantities. The 24-hour specimen should be measured and a comparison made between the amount ordinarily passed, and when an excess of water is being taken. The specific gravity is of much importance, if the amount of fluids ingested is considered. In addition, the condition of the heart, blood vessels, arterial tension, diet, intestinal canal, habits and past history should receive due attention.

Many patients with orthostatic albuminuria of renal origin will go for years without any evidence of nephritis, but our prognosis must not be made from the urine alone, but from the entire picture. Prudence demands that we be conservative, and yet not be too pessimistic and give patients unnecessary alarm about conditions which may not be dangerous. Success in discriminating between the insignificant forms of albuminuria, and those indicating renal disease, depends more upon eliminating nucleo-albumin and extrarenal albumin and albumose, than in any further refinement in our chemical examinations.

I realize that this paper has not cleared up the subject very much, but I will feel that it has served its purpose if it has assisted any of those present in looking upon albuminuria in a more judicial manner. It is important that we discard dogmatic statements and pre-conceived ideas that albuminuria necessarily means Bright's disease, and at the same time, it is of as much consequence that we be not too positive in our assertions that orthostatic or the so-called physiological albuminuria is always harmless.

### REFERENCES.

Bradshaw: *British Medical Journal, Nov. 24, 1906.*

Bruas: *International Medical Review, No. 1, 1907.*

Collier: *British Medical Journal, Jan. 5, 1907.*

Emerson: *Journal of the American Medical Association, January 6, 1906.*

Fox: *Lancet, August 5, 1906.*

Hastings: *Medical Record, July 7, 1906.*

Hastings and Hoobler: *American Journal of the Medical Sciences, February 1907.*

Heubner: *Berliner klinische Wochenschrift,* XLIV., *No. 1.*

Opie and Barker: *Journal of Experimental Medicine, March 14, 1907.*

Tunis: *American Journal of the Medical Sciences, July 1906.*

1014-15 CENTURY BUILDING.

